# Bacterial community composition of vermicompost-treated tomato rhizospheres

**DOI:** 10.1371/journal.pone.0230577

**Published:** 2020-04-06

**Authors:** Juana Munoz-Ucros, Kevin Panke-Buisse, Jamison Robe

**Affiliations:** 1 Horticulture Section, School of Integrative Plant Sciences, Cornell University, Ithaca, New York, United States of America; 2 US Dairy Forage Research Center, USDA ARS, Madison, Wisconsin, United States of America; Kyonggi University, REPUBLIC OF KOREA

## Abstract

Vermicompost application has been shown to promote plant growth, alter the rhizosphere microbiome, and suppress plant pathogens. These beneficial properties are often attributed to the activity of vermicompost-associated microorganisms. However, little is known about the microbial shifts that occur in the rhizosphere after vermicompost application. To better understand the impact of vermicompost treatments on the assembly of rhizosphere bacterial communities, 16S rDNA communities of vermicompost and rhizospheres of each peat- and soil-grown tomatoes were profiled after conventional fertigation, irrigation without additional nutrients, and addition of three different vermicompost-extracts. The full dataset consisted of 412 identified genera, of which 317 remained following stringent quality filtration. Tomato rhizosphere microbiome responses to treatments were complex and unique between peat and soil growth substrates. Direct colonization of vermicompost-origin taxa into rhizospheres was limited, with genera *Photobacterium* and *Luteimonas* colonizing peat rhizospheres, genera *Truepera*, *Phenylobacterium*, and *Lysinibacillus* colonizing soil rhizospheres, and genus *Pelagibius* appearing in both soil and peat rhizospheres. Further patterns of differential abundance and presence/absence between treatments highlight vermicompost-mediated effects on rhizosphere microbiome assembly as an interplay of rhizosphere medium, direct colonization of vermicompost-origin taxa and vermicompost-induced shifts in the rhizosphere microbial community. This exploratory analysis is intended to provide an initial look at 16S community composition of vermicompost and the effects of vermicompost treatment on the rhizosphere microbiome assembly to highlight interactions of potential merit for subsequent investigations.

## Introduction

Vermicompost (VC) is the product of digestive bio-oxidation of organic matter by certain species of worms and their digestive microbiota. Treatment of soils with VC can promote plant growth, alter the rhizosphere microbiome, and suppress plant pathogens. Plant growth promotion and resilience have been experimentally attributed to their physicochemical properties (buffering capacity, air/water holding capacity, plant growth regulators, nutrient forms, etc.) and their active microbiome [1–5]. Application of vermicompost has conferred benefits in a wide variety of crops including agronomic [6,7], horticultural [1,8–10], and timber [11]. Furthermore, extracts and teas made from VC exhibit similar effects on plant growth, but do not impart the same physical properties as vermicompost amendment [12,13]. The specificity and degree of effect vary greatly between crop species and VC variety [14,15].

There is high demand for microbially-active soil amendments for field and greenhouse production, and VC is a sustainable, versatile solution. In addition, vermicomposting has been used for the creation of value-added product from high-volume byproducts of the wine and animal agricultural industries [16–18]. VC has been demonstrated to enhance plant growth and elicit changes in the soil microbiome of treated plants in greenhouse and field conditions [19]. They can be made from a wide variety of substrates that may otherwise be waste material, and the process generates worm biomass which can also be harvested as livestock feed or a product for sale [20,21].

Despite the body of literature supporting the growth-promoting effects of VC, less is known about its microbiome or the rhizosphere microbial community shifts following its application into plant growth media. Traditional work centered around cultured strains, mycorrhizae, and non-sequence-based methods of assessing microbial community structure [4]. More recently, sequencing-dependent analysis have become available that investigate changes in the microbiome during composting [22] and pathogen-vermicompost interactions [23]. Here, we investigate the impact of VC-extract treatments on bacterial microbiome assembly in each peat- and soil-grown tomato rhizospheres. The objective of this work is to begin to disentangle the complex interplay of biotic and abiotic factors that influence vermicompost-related changes in plant growth by focusing on rhizosphere microbiome assembly. Comparison of control rhizosphere communities to those treated with intact and heat-killed vermicompost offers a framework for highlighting potential taxa of interest within the vermicompost as well as native rhizosphere taxa responding to vermicompost-origin taxa or metabolites. Control rhizospheres were fertigated or irrigated and VC-treated rhizospheres received one of three VC-extracts: unaltered VC-extract (UVC), sterilized VC-extract (SVC), or sterilized, reinoculated VC-extract (RVC).

## Materials and methods

### Tomato rhizosphere sample preparation and collection

Tomatoes were greenhouse-grown at Cornell University and rhizospheres in each treatment were sampled at 8, 12, and 16 weeks of age. The earliest time point was chosen to allow full development of the tomato rhizospheres, while the 12 and 16 week timepoints corresponded to the start of flowering and fruit development and were chosen to capture potential shifts related to plant developmental stage. Peat potting media consisted of three parts peat (fertilizer-free, Lambert Peat Moss, Riviere-Ouelle, QC, Canada), one part perlite, and lime at a rate of 2.225g/L for pH adjustment. For the soil medium, soil from a tilled corn field, was collected in September of 2013 at Cornell University (Ithaca, NY), dried and sieved to 4mm. Dried, sieved soil was mixed 1:1 with a 3:1 mixture of sand and commercially available topsoil (Agway, Ithaca, NY) sourced from Pennsylvania, USA in a soil mixer. The resulting soil mixture was then mixed 3:1 with perlite to further improve drainage and reduce pot weight. The final soil medium consisted of approximately 37.5% field soil, 28.125% sand, 25% perlite, and 9.375% topsoil.

Control (C) pots received only water irrigation. Fertigated (F) pots received a weekly addition of 25mL of 100ppm 20-10-20 Jack’s Professional General Purpose Fertilizer (J.R. Peters, Inc., Allentown, PA, USA). VC (NPK 1.5–0.7–1.5) samples were provided by Wormpower (Avon, NY, USA) and were produced from corn silage and dairy manure that was first thermophilically composted before vermicomposting. All VC-extracts were made from the same 15lb bag of VC stored at room temperature and were made by suspending VC solids in autoclaved deionized water then shaking for 2.5h at 150rpm. The slurry was filtered through double nylon mesh and the solids were discarded. This VC extract (UVC) was used directly at 10% v/v, based on preliminary trials, for the UVC tomato pots. The sterilized VC (SVC) treatment was prepared by extracting VC that had been autoclaved twice over a 3-day period (liquid cycle, 35 min) and left at room temperature in sealed autoclave bags between autoclave treatments and applying to tomato pots at 10% v/v. To create the sterilized, reinoculated VC (RVC) treatment, UVC was centrifuged for 35min at 3000rcf and the resulting pellet was resuspended in phosphate-buffered saline (PBS). The PBS solution was combined 1:9 with SVC immediately prior to planting and this RVC extract was added to tomato pots at 10% v/v. All VC extracts were applied to the potting medium surface at seeding. Tomato seeds were started in flats containing sterile perlite. At 4 weeks, tomato seedlings and intact rhizospheres were transplanted into 5 4” diameter pots containing peat or soil per treatment. Rhizospheres were destructively harvested at three 4-week intervals from each treatment by removing the topmost centimeter of soil and root mass on all sides and sieving to remove large roots. S1 Fig illustrates the experimental design used (S1 Fig). Remaining soil was homogenized and stored at -20°C prior to DNA extraction.

### DNA extraction, amplification, sequencing, and analysis

Vermicompost and rhizosphere DNA was extracted from frozen samples using the PowerSoil DNA Isolation Kit (MO BIO Laboratories, Inc., Carlsbad, CA, USA). Approximately 0.15 g of sample was used for isolation of DNA. The vermicompost extract was prepared by filtering 10mL of extract through a 0.22 micrometer filter and placing the whole filter in the extraction kit. Quantification was performed with the standard dsDNA quantification protocol for Picogreen (Thermo Fisher Scientific, Inc., Waltham, MA, USA) to ensure all extracted samples contained greater than 10ng DNA/microliter in solution. Extracted samples falling below this threshold were extracted again. All pipetting for DNA extraction was conducted with an Eppendorf epMotion 5075 pipetting robot (Eppendorf AG, Hamburg, Germany). VC samples were amplified in triplicate with PCR primers detailed in Caporaso et al. (2012) that target the bacterial/archaeal 16S rRNA gene variable region 4 (515 F/806 R) for downstream paired-end Illumina MiSeq (Illumina, Inc., San Diego, CA, USA) barcoded sequencing [24]. Rhizosphere samples were amplified in duplicate with the dual-indexed primers detailed in Kozich et al., 2013 [25]. VC PCR amplicons were quantified and 200ng of each sample were pooled and purified with the desalting protocol of the Qiagen QiaQuick spin filter purification kit (QIAGEN Inc., Valencia, CA, USA). All PCR reactions contained 25ng template DNA. Rhizosphere PCR amplicons were quantified and 200ng of each sample were pooled and purified with the HighPrep magnetic beads (MagBio Genomics, Gaithersburg, MD, USA). Amplicon pools were submitted separately to the Cornell Institute of Biotechnology Sequencing Facility (Ithaca, NY, USA) with the sequencing primers specific to the primers used for amplification of the 16S V4 region.

Paired-end reads were trimmed to remove the first 25 bases in the forward reads and first 10 bases in the reverse reads in the R package dada2 [26]. Reads were then truncated at the first low-quality base and quality-filtered to remove those with ambiguous bases, an average quality score below 25, or fewer than 250nt. Paired-end sequences passing quality filtration were merged, resulting in 39159 sequence variants in the full dataset, which dropped to 15675 variants following chimera removal, and to 12576 variants after collapsing length variants into single representative variants. The 6442 sequences remaining following low-abundance filtration (single/double/triple-ton removal) and merging were assigned taxonomy from the Greengenes 13_8 reference database, and analyzed with the R package phyloseq. [27] Due to the differences in primers, sequencing runs and persisting sequence length variation between the VC samples and rhizosphere samples, the dataset was agglomerated based on taxonomy, which collapsed sequence variants into single examples of each taxon at the genus level. Operational taxonomic units (OTU) not annotated to the genus level were removed. After agglomeration and quality control, the dataset consisted of 317 OTUs identified at the genus level. All data files are available from the Qiita database (ID 11929 and at URL: https://qiita.ucsd.edu/study/description/11929).

The DESeq2 package was used for estimation of differential abundance with an alpha-value of 0.05 and Benjamini-Hochberg adjusted p-values [28]. Significance of differences in presence and absence between groups was performed by adonis in the vegan package for R at and alpha-level of 0.05. Venn diagrams were produced with the Bioinformatics & Evolutionary Genomics Venn diagram tool at http://bioinformatics.psb.ugent.be/webtools/Venn/. All other figures were generated with the ggplot2 R package.

## Results and discussion

This dataset provides an initial look at 16S community composition of VC and the effects of VC treatment on the rhizosphere bacterial community. The analysis presented is exploratory and intended to highlight interactions of potential merit for future investigations.

VC bacterial community compositions differed significantly from water extracts derived from VC solids (S2 Fig). Alpha diversity (Shannon Diversity Index) varied widely between time points for VC-extract, but was more uniform in VC solids. In the VC-extracts, *Verrucosispora* and *Actinomadura* genera showed reduced abundance, while *Lactococcus*, *Bacillus*, and *Hyphomicrobium* were enriched (S3 Fig). Shifts were attributed to the extraction process, which combines VC solids in water. The dilution and shift to a primarily aqueous environment after the addition of water represents a strong ecological filter and may compound existing sample-to-sample variation within the composting process.

VC-treated and fertigated tomato rhizospheres responded differently with plant age and between peat and soil (S4 Fig). The patterns of bacterial community composition and abundance across groups were complex and showed clear and convoluted differences between treatments and peat/soil rhizospheres (S5 Fig).

**Fig 1:**
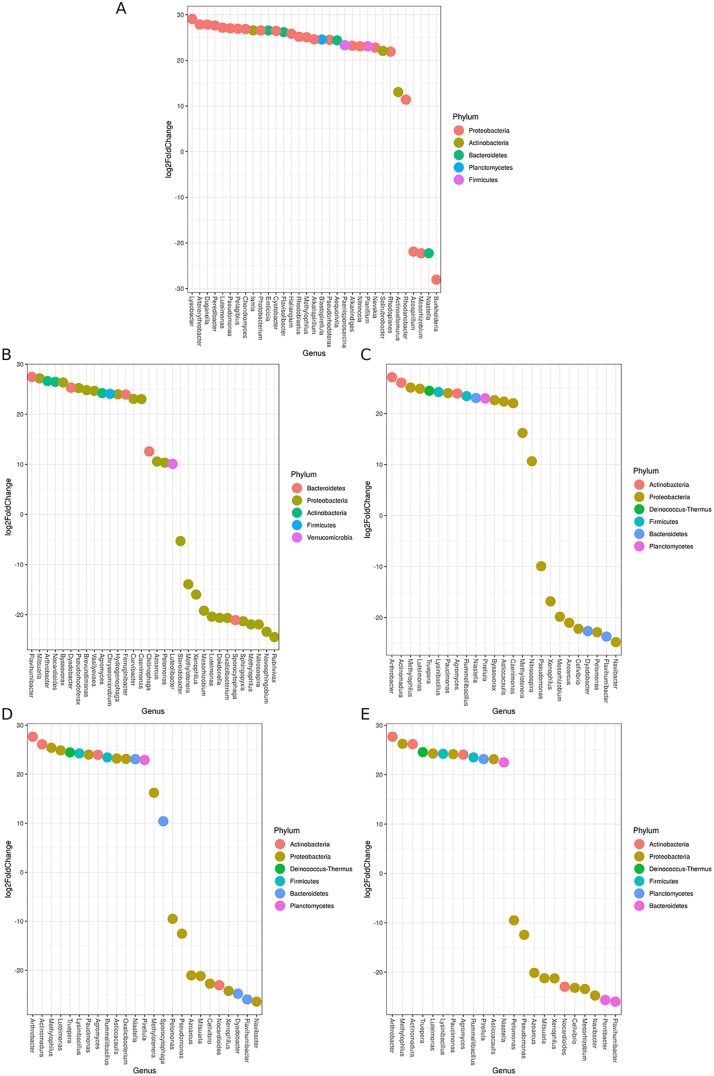
Log (2-fold) change of significantly differing genera between rhizosphere treatments. DESeq2 calculated differential abundance of significant (Benjamini-Hochberg adjusted p<0.05) genera between (A) UVC and irrigated peat rhizospheres, (B) UVC and irrigated soil rhizospheres (C) fertigated and irrigated soil rhizospheres, (D) RVC and UVC soil rhizospheres, (E) SVC and UVC soil rhizospheres. Positive values represent an enrichment of the taxon in the first-listed treatment as compared to the second, i.e., in (A), positive values indicate that the taxa are more abundant in UVC peat rhizospheres than in irrigated. Colors correspond to the phylum while genera are listed on the x-axis.

Treatment with VC extracts produced significant shifts in the rhizosphere microbial community abundance that contrasted with fertigated and irrigated control rhizospheres. In peat, significant changes in taxonomic abundance were identified only between the irrigated and UVC-treated rhizospheres (Fig 1A). UVC-treated peat rhizospheres contained significantly less *Burkholderia*, *Mesorhizobium*, and *Azospirillum* than irrigated control rhizospheres, and significantly more *Lysobacter*, *Pseudomonas*, and *Peredibacter*. The increase of N-fixing genera in the control rhizospheres relative to the UVC treatment is likely an enrichment due to low added N. Genus *Pseudemonas* has been frequently observed in plant growth-promotion and genus *Lysobacter* is a rich taxonomic source of antimicrobials, both of which align with reports of pathogen suppression and enhanced plant growth associated with VC application. In soil, sterilized VC-extract (SVC) and sterilized-reinoculated VC-extract (RVC) treated rhizospheres displayed similarities to the UVC microbiome by presence/absence as well as unique effects on rhizosphere community structure. Differential abundance analysis confirmed significant shifts in taxonomic abundance that were unique to each treatment, but also several taxonomic shifts that were mirrored when comparing the irrigated control and fertigated rhizospheres (Fig 1B–1E). In particular, RVC, SVC, and fertigated rhizospheres exhibited significant reductions in *Naxibacter* and *Azoarcus* and increases in *Actinomadura* and *Lysinibacillus* when compared to UVC and irrigated rhizospheres respectively. This may point to an effect of added nutrients, regardless of source, on relative abundance.

**Fig 2:**
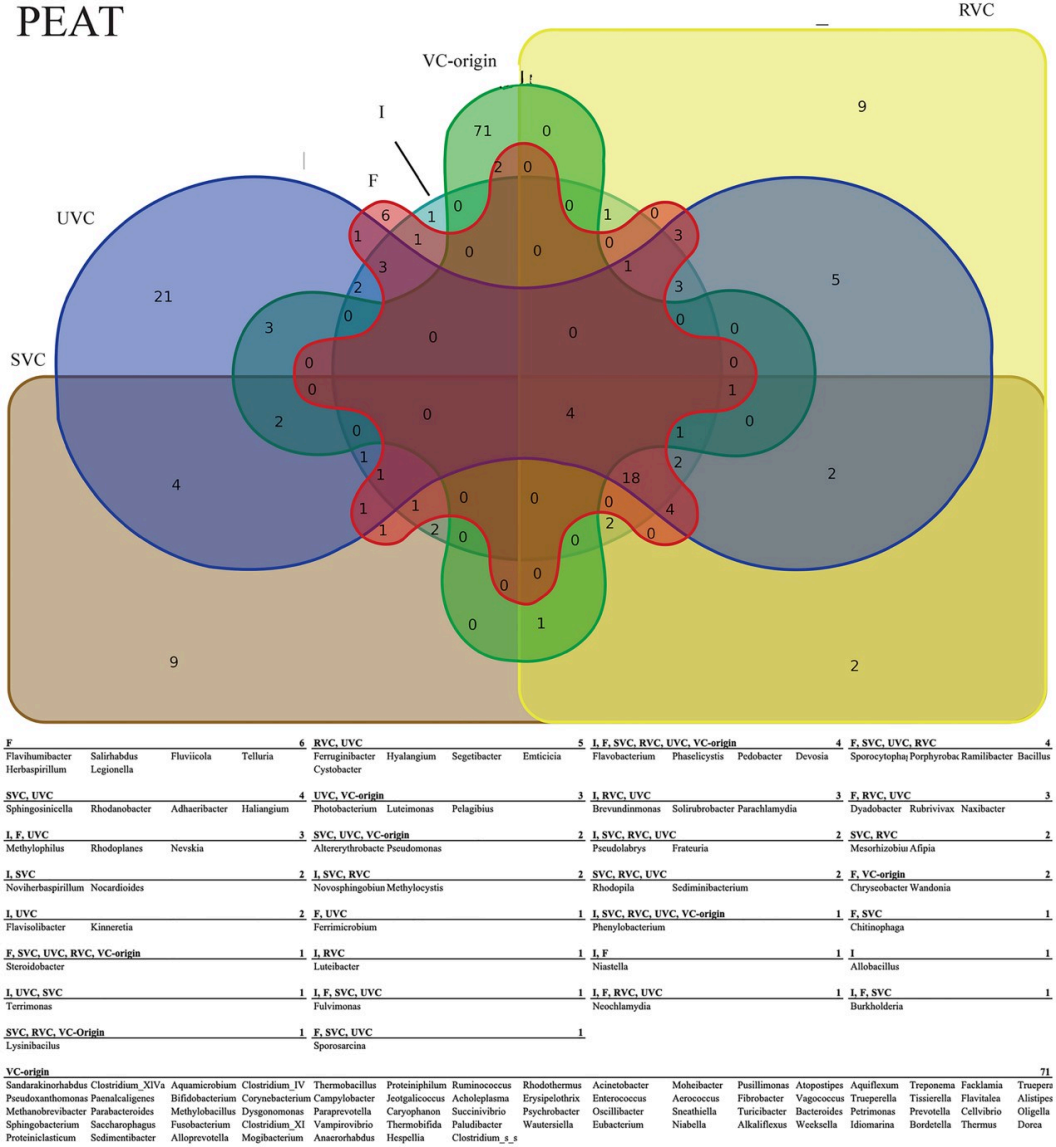
Peat rhizosphere taxa diagram. Venn diagram of observed genera across all treatments in peat. The associated table lists genera corresponding to treatment-specific and shared taxa.

**Fig 3:**
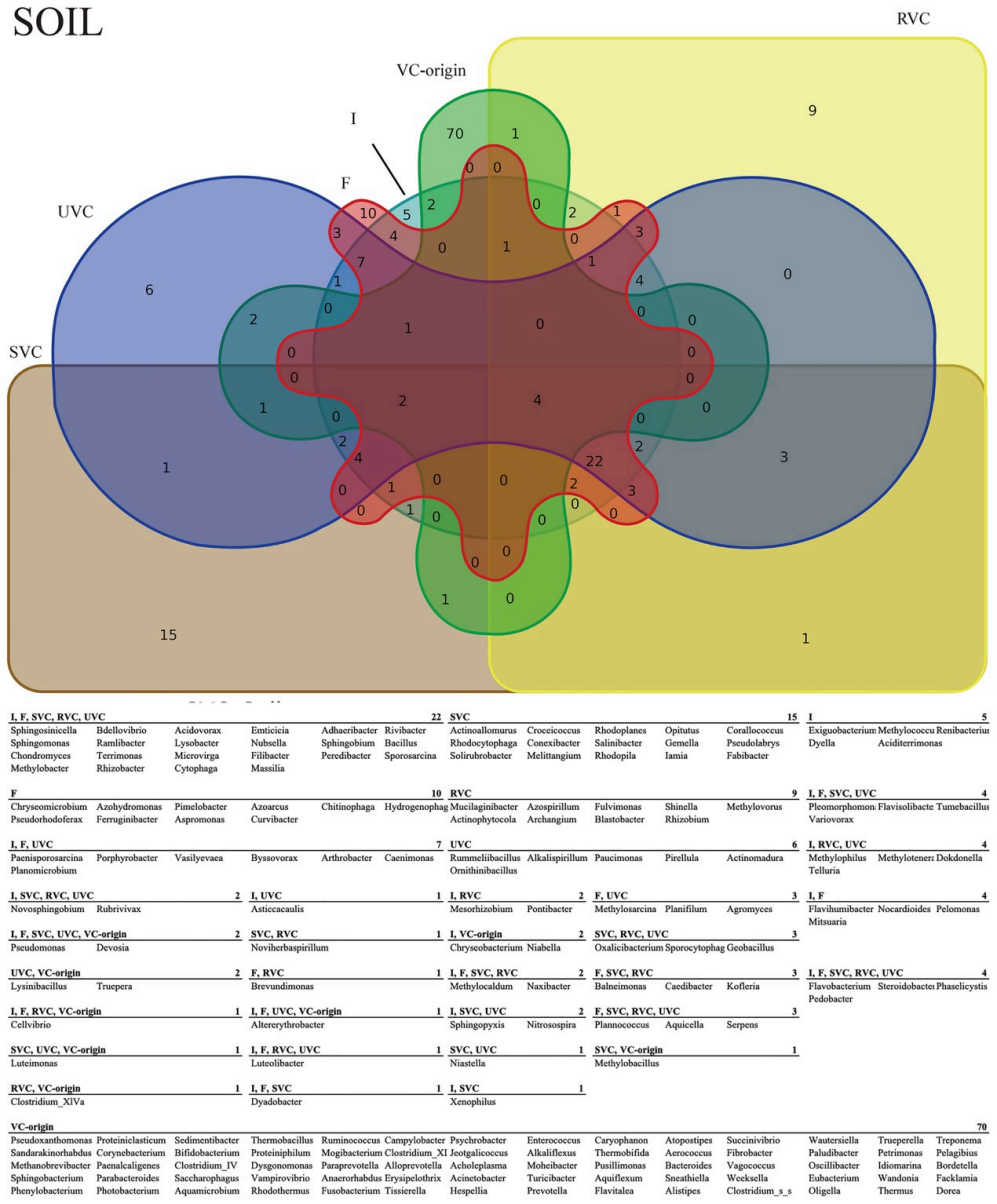
Soil rhizosphere taxa diagram. Venn diagram of observed genera across all treatments in soil. The associated table lists genera corresponding to treatment-specific and shared taxa.

**Fig 4:**
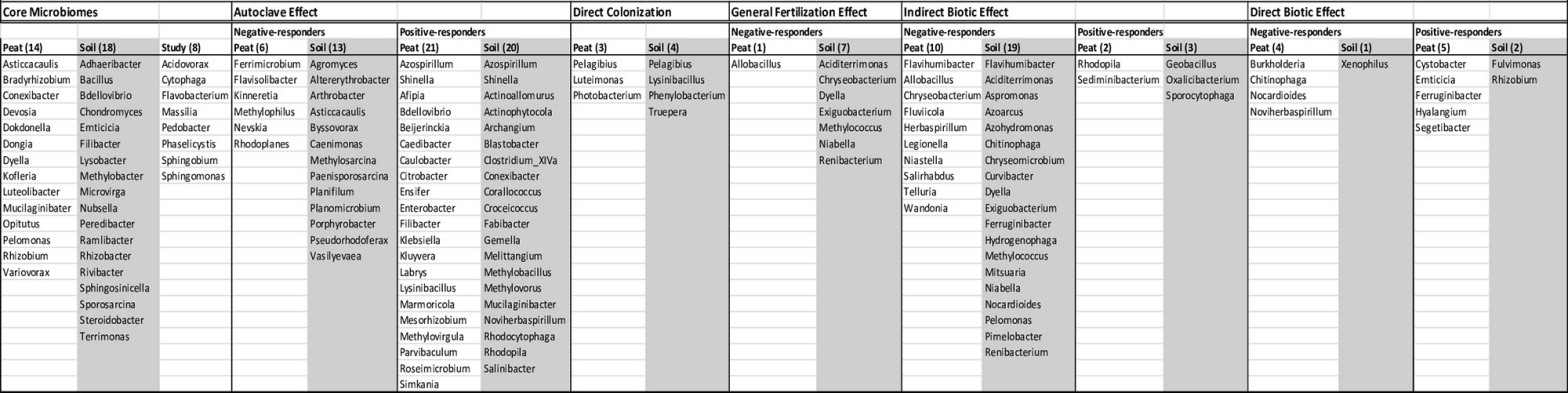
Potential patterns of taxa presence/absence across treatments. Summary of genus presence/absence across treatments grouped by potential response bin. Soil rhizosphere taxa are shaded in gray. Genera present in both peat and soil rhizospheres for a given bin are listed first with all other genera listed alphabetically thereafter.

While significant shifts in relative abundance were comparatively few, observed trends in bacterial presence/absence were numerous and complex. Binning taxa into groups or effects based on their patterns of appearance across treatments as represented by Venn diagram allowed us to highlight potentially important taxa in the dataset (Figs 2 and 3). For example, taxa present in both the fertigated and sterilized vermicompost treatment rhizospheres, but not the irrigated or unaltered vermicompost rhizospheres are likely responding to additional nutrients rather than specific biotic components of the vermicompost treatments. The bins explored include core microbiomes, direct colonization of VC-origin taxa, general fertilization effects, autoclaving effects, direct biotic effects, and indirect biotic effects. The criteria for membership in each bin are discussed below and the taxa of note from these bins are summarized in Fig 4.

The whole-study core microbiome was represented by genera present in all treatments in both soil and peat, while the soil and peat core microbiomes were considered those taxa present in all treatments of either soil or peat, but not the other (S6 Fig). Eight taxa, including *Flavobacterium*, *Pedobacter*, *Phaselicystis*, and *Sphingomonas* were present in all samples, indicating general ubiquitous rhizosphere members. Eighteen taxa were in the soil rhizosphere core including *Lysobacter*, *Methylobacter*, *Terrimonas*, *Rhizobacter*, and *Steroidobacter*. Fourteen taxa make up the peat rhizosphere core including *Luteolibacter*, *Bradyrhizobium*, *Rhizobium*, *Devosia*, and *Pelomonas*.

VC-origin taxa that appeared in UVC treated rhizospheres, but not in irrigated or fertigated rhizospheres, were the criteria we used to identify potential direct colonization (S7 Fig). Of the 85 observed VC-origin taxa, eighteen appear in the tomato rhizospheres. Six of these are unique to UVC treatments, indicating that the other 8 are likely co-occurring due to ubiquity rather than colonization. VC-origin genera *Photobacterium* and *Luteimonas* are exclusively present in UVC peat rhizospheres and genera *Truepera*, *Phenylobacterium*, and *Lysinibacillus* are unique to the UVC soil rhizospheres. VC-origin genus *Pelagibius* appears in both soil and peat UVC rhizospheres.

Our criterion for identifying taxa responding to a general fertilization effect was mutually-exclusive membership in the irrigated control, which received no added nutrients, and all other treatment rhizospheres, which received non-zero amounts of added nutrients (S8 Fig) For peat rhizospheres, genus *Allobacillus* is the only taxon unique to the irrigated control, and, in soil, 7 genera were unique to irrigated rhizospheres. These genera may indicate taxa that are selected against by nutrient addition to the rhizosphere. Many genera, 77 in peat rhizospheres and 60 in soil rhizospheres, were associated with the nutrient-addition treatment rhizospheres and represent potential general fertilization responders. Forty-three and 61 genera, respectively in peat and soil rhizospheres, were shared between the irrigated and nutrient-added groups.

In order to identify potential responses to autoclaving associated with the SVC and RVC rhizospheres, taxa were binned into three groups: UVC, irrigated/fertigated, and SVC/RVC (S9 Fig). Twenty-one and 20 genera in peat and soil rhizospheres, respectively, were unique to the SVC/RVC, or “autoclaved” group, indicating a potential group of autoclaved treatment enrichment-responders in the dataset. Six genera in peat rhizospheres and thirteen genera in soil rhizospheres were shared between the irrigated/fertigated and UVC groups, but absent in the autoclaved group, identifying a pool of potential autoclaved treatment negative-responders. In the absent “negative”-responders groups, none of the genera were shared between soil and peat rhizospheres, while in the “positive”-responders, *Azospirillum* and *Shinella* appeared in peat and soil rhizospheres.

Direct biotic effects were defined as being associated with treatments that contained living microbes, *i*.*e*. UVC and RVC, as compared to those treatments with no living biotic component. (S10 Fig) In peat, 5 genera were associated with the UVC/RVC biotic group and 4 genera were absent in UVC/RVC, but present in the I/F/SVC group. In soil, *Fulvimonas* and *Rhizobium* were unique to the UVC/RVC group and *Xenophilus* was absent. These patterns may highlight taxa sensitive to the presence of the live cells contained in VC treatments, even at the low-cell density of the RVC treatment.

Similarly to the direct biotic effects, we also sought to outline potential indirect biotic effects, which were defined as changes associated that could be attributable to residual molecules of microbial origin. These were highlighted by comparing responses shared by UVC, RVC, and SVC to those common to irrigated and fertigated treatments (S7 Fig). In peat rhizospheres, *Rhodopila* and *Sediminibacter* were present in UVC, RVC, and SVC, but absent in irrigated or fertigated rhizospheres. In soil rhizospheres, *Oxalicibacterium*, *Geobacillus*, and *Sporocytophaga* were present in UVC, RVC, and SVC, but absent in irrigated or fertigated rhizospheres. Taxa present in both irrigated and fertigated rhizospheres, but absent from all VC treatments numbered 10 in peat rhizospheres and 19 in soil rhizospheres, with *Flavihumibacter* as the only shared genus.

## Conclusions

The presented data suggest that VC-mediated effects on the rhizosphere microbiome can be a complex interplay of rhizosphere medium, direct colonization of VC-origin taxa, VC taxa-induced shifts in the rhizosphere community, and indirect alteration of the rhizosphere community via secondary metabolites or nutrients that remain after autoclaving. The observation of limited direct colonization, and the distinct rhizosphere media preference of colonizing taxa, decreases the search space for novel beneficial vermicompost taxa. Indirect alterations of rhizosphere microbiome assembly by vermicompost treatments, both in sterilized and unaltered vermicompost were clearly highlighted, but the consequences of these shifts are beyond the scope of the current study. Enrichment of *Lysobacter*, a genus known for antimicrobial production, and *Pseudemonas*, a genus frequently observed as promoting plant growth, support observations of vermicompost-attributed plant growth promotion and plant disease suppression. Rhizosphere microbial community and its response to vermicompost addition was largely influenced by age. A better understanding of the microbiome-derived benefits of VC is a necessary step in the development of commercial and field applications to support sustainable agriculture and soil health. Future work probing the role of the microbial community in VC-derived plant growth promotion and disease suppression should include both fungal and bacterial communities.

## Supporting information

S1 Fig Diagram of the experimental treatments. Illustration of the treatment groups, replication, and rhizosphere ages.
(TIF)

S2 Fig Relative abundance of bacterial phyla in vermicompost and vermicompost extract. Relative abundance of bacterial phyla in vermicompost and vermicompost extract. (A) Relative abundance of individual time points. (B) Sum of relative abundances within VC and VC-extract (VE).
(TIF)

S3 Fig Log (2-fold change) of significantly differing genera between vermicompost solids and vermicompost extract. DESeq2 calculated differential abundance of significant (Benjamini-Hochberg adjusted p<0.05) genera between vermicompost solids and vermicompost exrtract. Colors correspond to phylum while genera are listed on the x-axis.
(TIF)

S4 Fig Relative abundance of bacterial phyla in soil and peat tomato rhizosphere. (A) Relative abundance of bacterial phyla peat and soil tomato rhizospheres. (B) Relative abundance of bacterial phyla in peat and soil tomato rhizospheres separated by plant age.
(TIF)

S5 Fig Principal coordinates analysis of unweighted unifrac distances. (A) Principal coordinates plot of unweighted unifrac distance of tomato rhizospheres. (B) Principal coordinates plot of weighted unifrac distance of tomato rhizospheres. Peat rhizospheres are represented by circles, soil rhizospheres by triangles. Point colors represent Irrigated (C-red), Fertigated (F-brown), Reinoculated vermicompost (RVC-green), Sterilized vermicompost (SVC-blue), and Vermicompost (VC-purple) treatments.
(TIF)

S6 Fig Peat and soil rhizosphere core microbiomes. Venn diagram of observed genera across all treatments in peat and soil rhizospheres. The associated table lists genera corresponding to peat-specific, soil-specific, and shared taxa.
(TIF)

S7 Fig Direct colonization of VC-origin taxa in peat and soil rhizospheres. Venn diagram of observed genera across treatments to highlight the direct colonization of A) peat and B) soil rhizospheres by vermicompost-origin taxa. The associated tables list genera corresponding to the groups delineated by the diagram.
(TIF)

S8 Fig General fertilization response in peat and soil rhizospheres. Venn diagram of observed genera across treatments to highlight potential general fertilization responses of A) peat and B) soil rhizospheres to VC treatments. The associated tables list genera corresponding to the groups delineated by the diagram.
(TIF)

S9 Fig Autoclave effect of VC treatments in peat and soil rhizospheres. Venn diagram of observed genera across treatments to highlight potential autoclaving effects of A) peat and B) soil rhizospheres to VC treatments. The associated tables list genera corresponding to the groups delineated by the diagram.
(TIF)

S10 Fig Biotic effect of VC treatments in peat and soil rhizospheres. Venn diagram of observed genera across treatments to highlight potential direct and indirect biotic effects of A) peat and B) soil rhizospheres to VC treatments. The associated tables list genera corresponding to the groups delineated by the diagram.
(TIF)
