# Supplementary figures and images for: Bacterial community composition of vermicompost-treated tomato rhizospheres
Source: PLoS One. 2020 Apr 6;15(4):e0230577. doi: 10.1371/journal.pone.0230577 (PMC7135065; doi:10.1371/journal.pone.0230577)

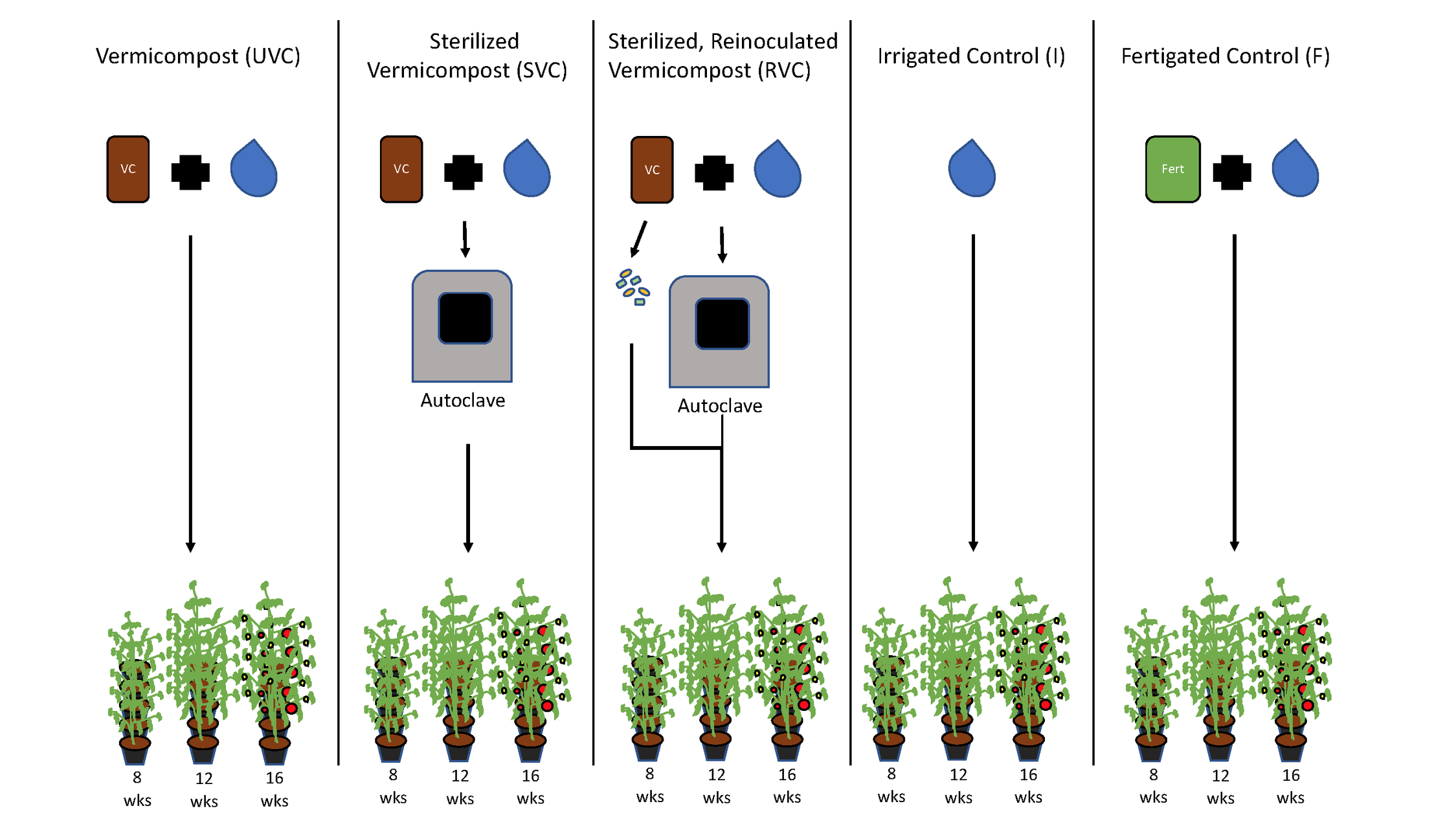

Supplement: S1 Fig — Illustration of the treatment groups, replication, and rhizosphere ages. (TIF) [file pone.0230577.s001.tif]

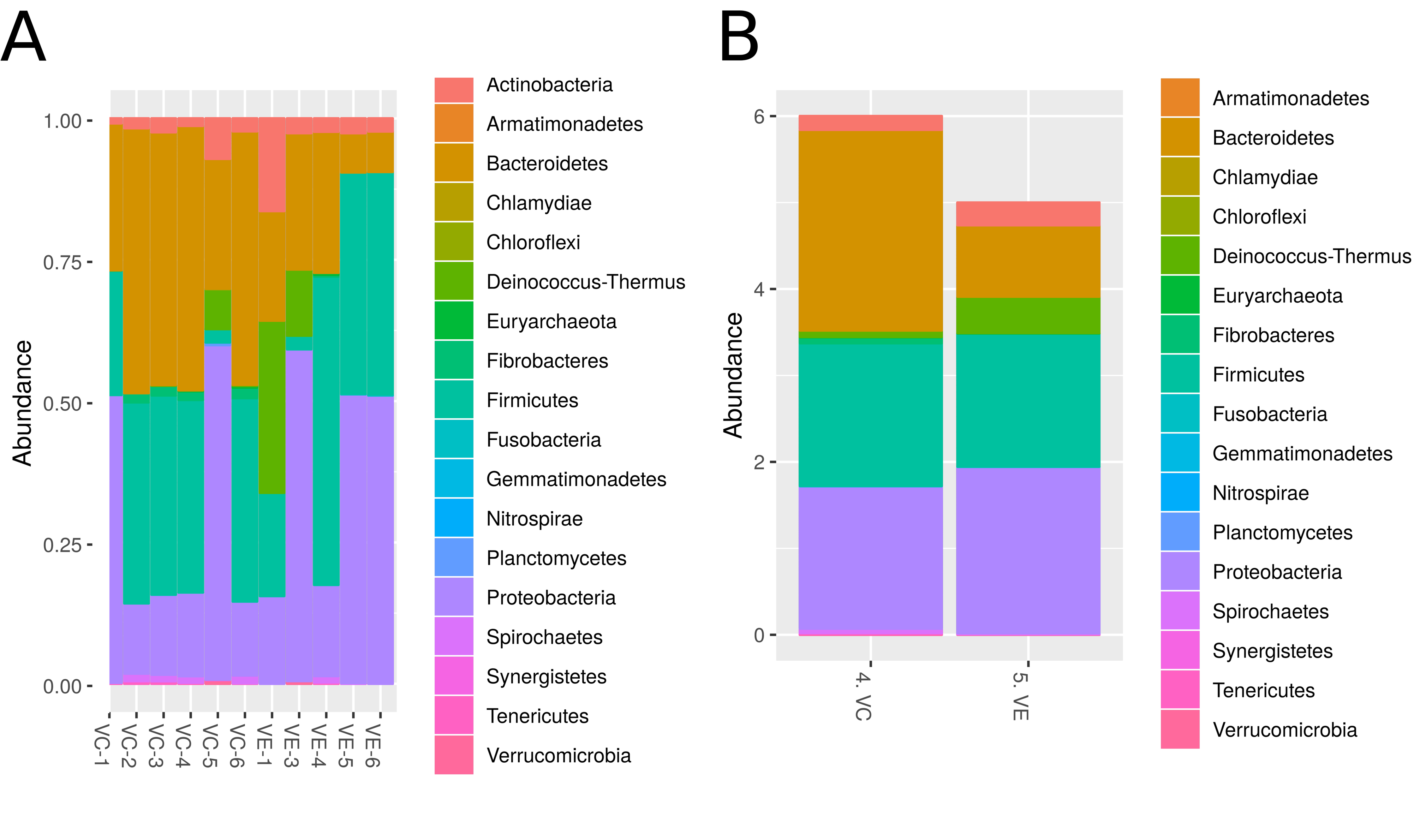

Supplement: S2 Fig — Relative abundance of bacterial phyla in vermicompost and vermicompost extract. (A) Relative abundance of individual time points. (B) Sum of relative abundances within VC and VC-extract (VE). (TIF) [file pone.0230577.s002.tif]

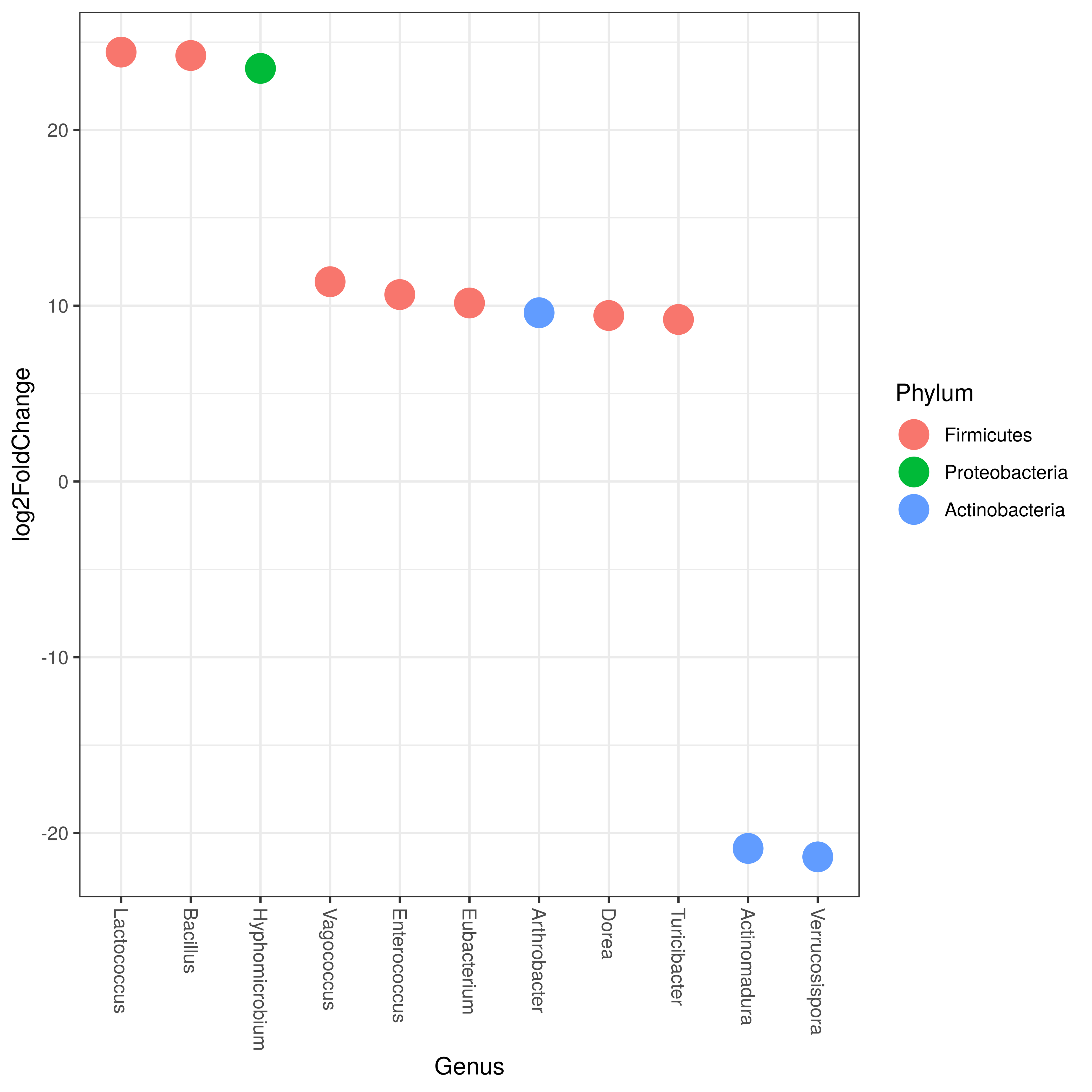

Supplement: S3 Fig — DESeq2 calculated differential abundance of significant (Benjamini-Hochberg adjusted p<0.05) genera between vermicompost solids and vermicompost exrtract. Colors correspond to phylum while genera are listed on the x-axis. (TIF) [file pone.0230577.s003.tif]

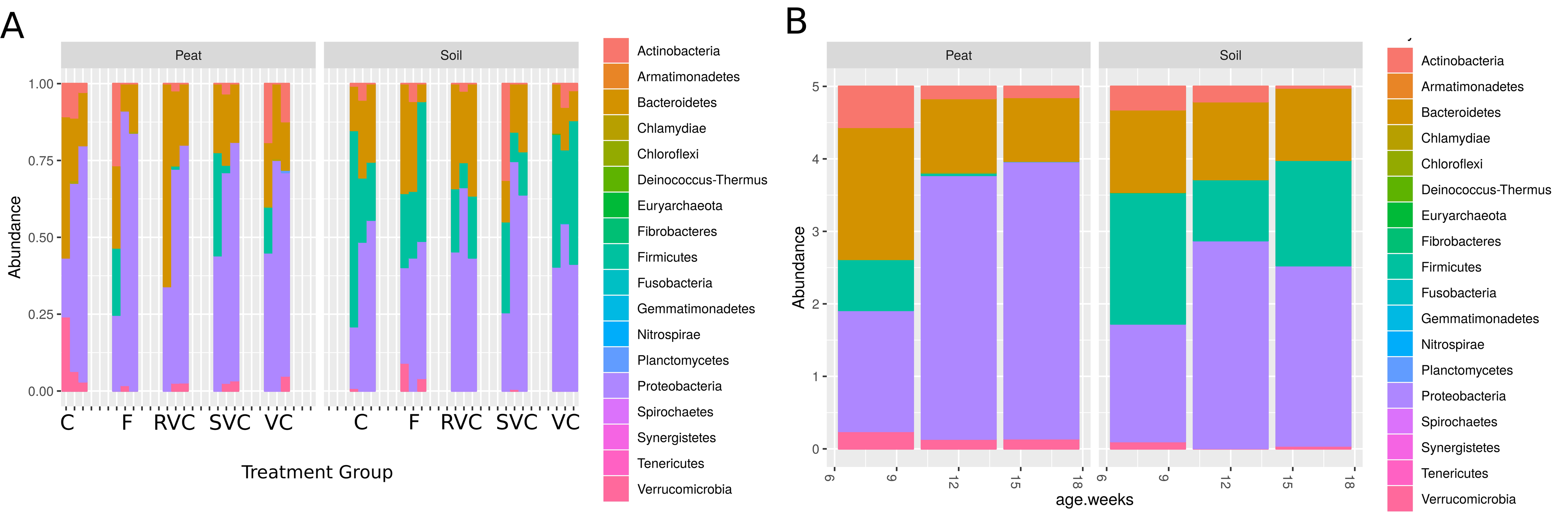

Supplement: S4 Fig — (A) Relative abundance of bacterial phyla peat and soil tomato rhizospheres. (B) Relative abundance of bacterial phyla in peat and soil tomato rhizospheres separated by plant age. (TIF) [file pone.0230577.s004.tif]

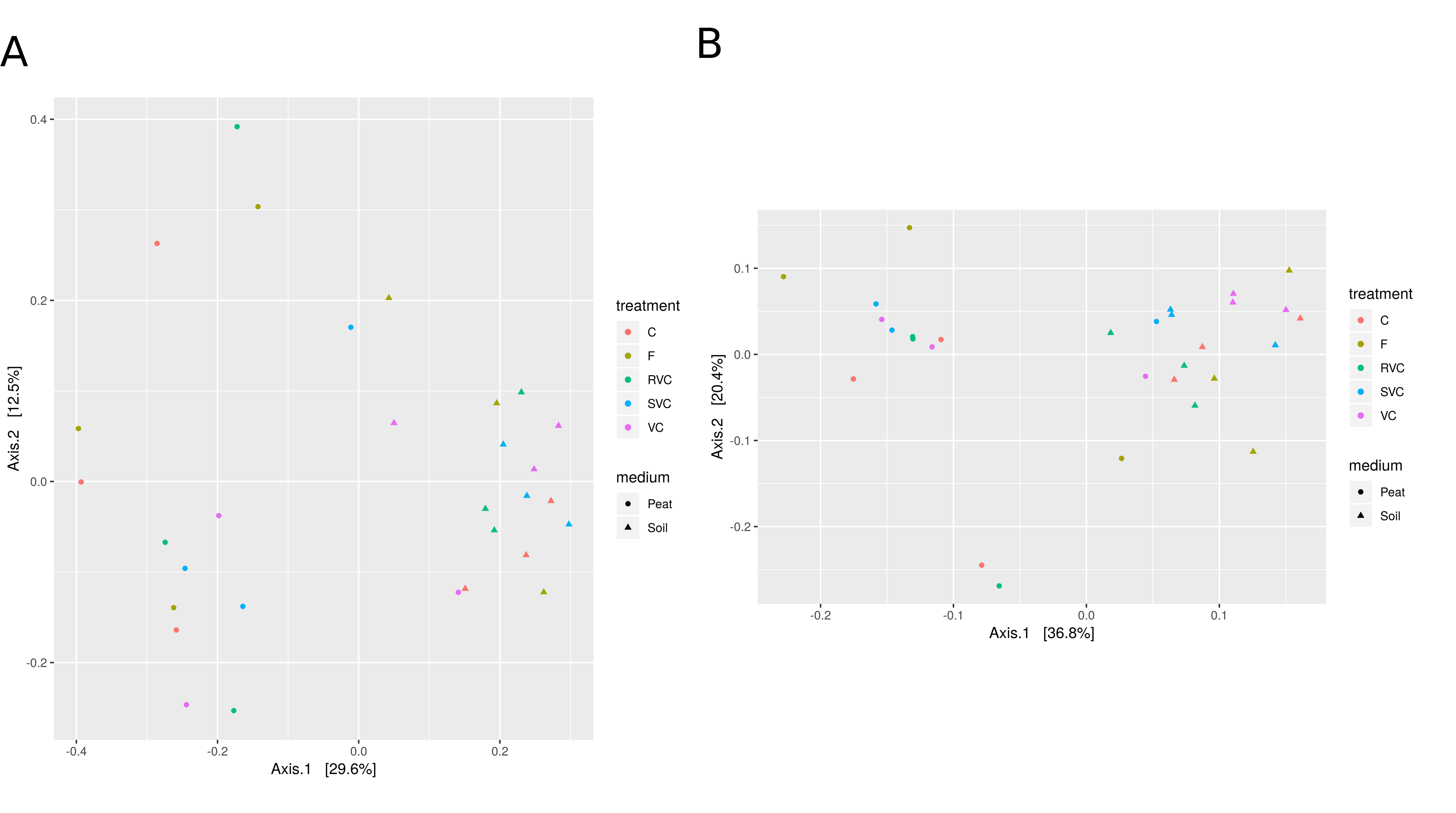

Supplement: S5 Fig — (A) Principal coordinates plot of unweighted unifrac distance of tomato rhizospheres. (B) Principal coordinates plot of weighted unifrac distance of tomato rhizospheres. Peat rhizospheres are represented by circles, soil rhizospheres by triangles. Point colors represent Irrigated (C-red), Fertigated (F-brown), Reinoculated vermicompost (RVC-green), Sterilized vermicompost (SVC-blue), and Vermicompost (VC-purple) treatments. (TIF) [file pone.0230577.s005.tif]

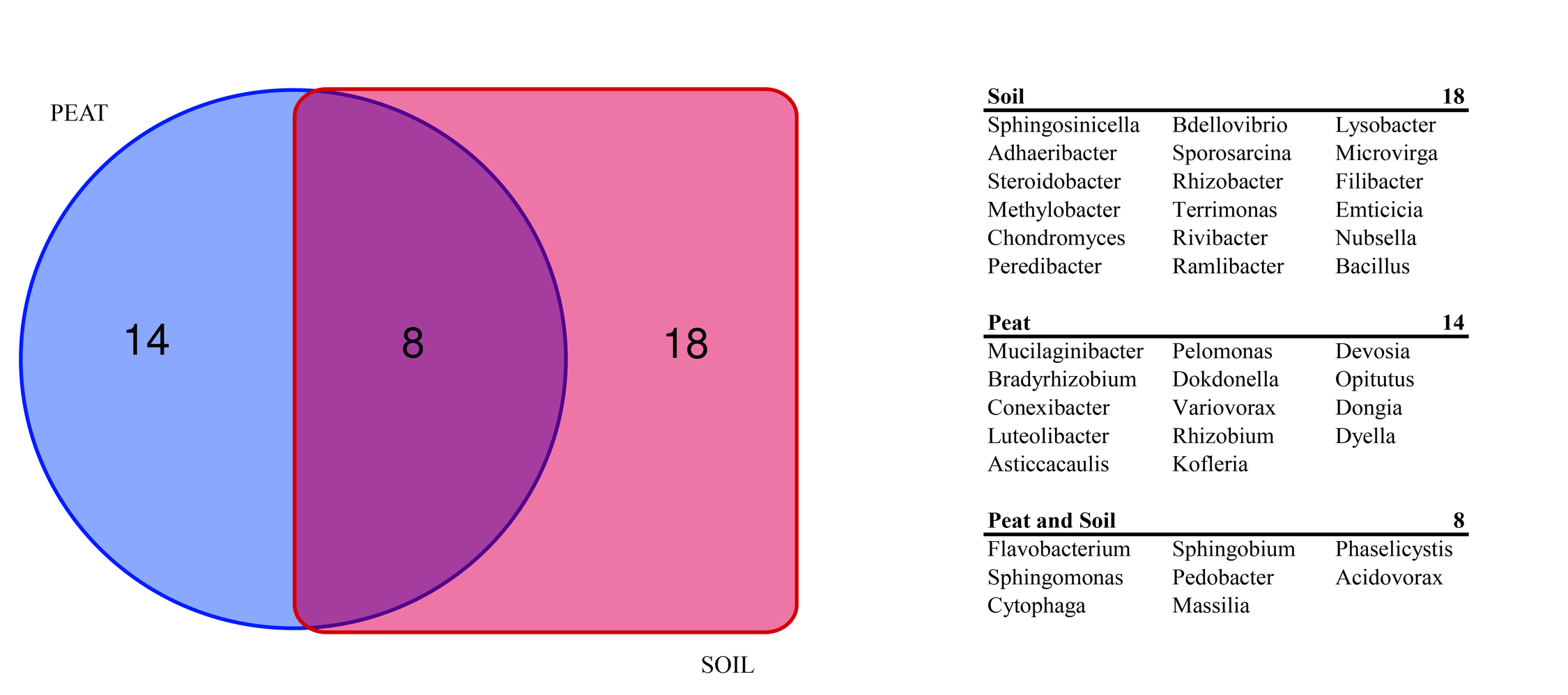

Supplement: S6 Fig — Venn diagram of observed genera across all treatments in peat and soil rhizospheres. The associated table lists genera corresponding to peat-specific, soil-specific, and shared taxa. (TIF) [file pone.0230577.s006.tif]

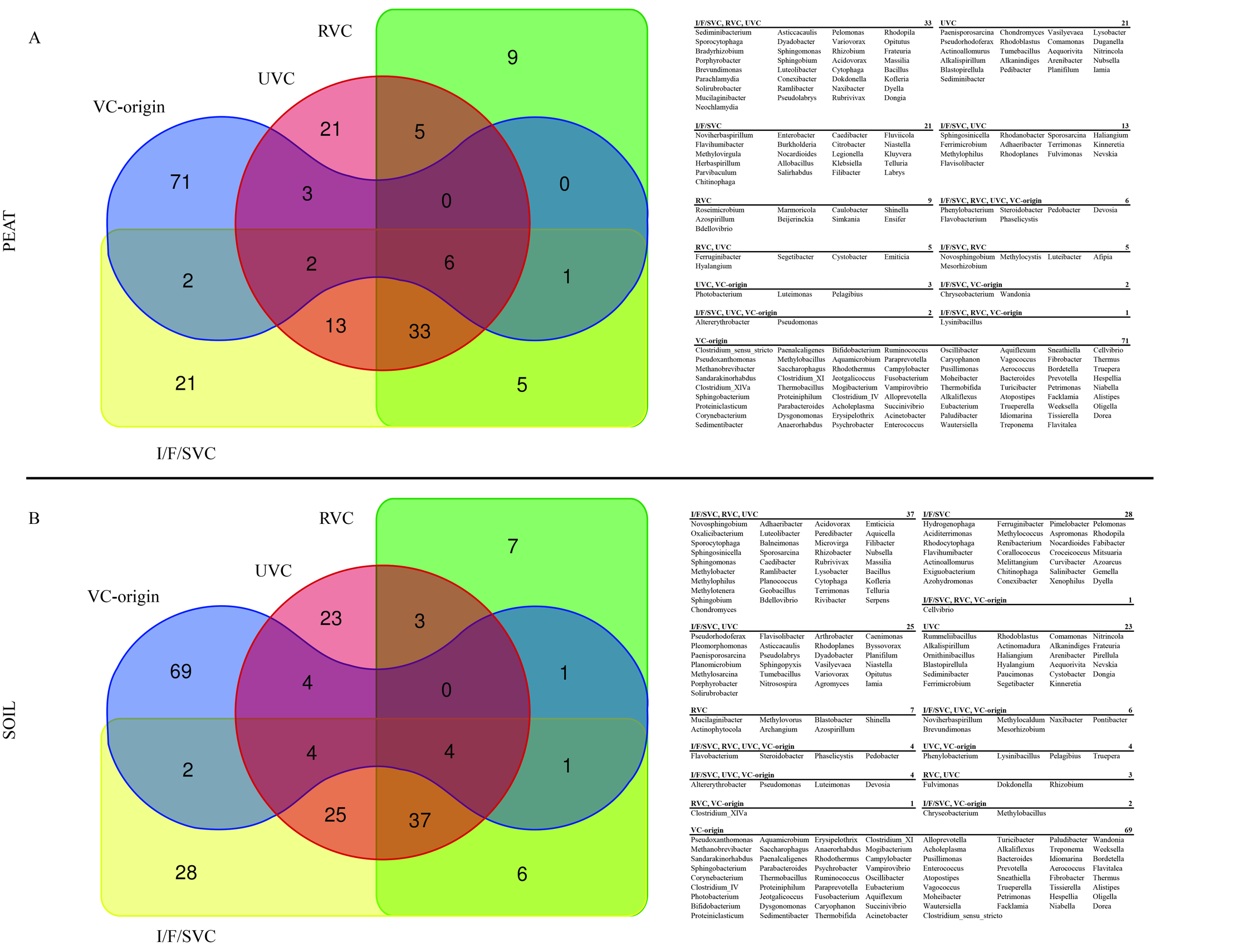

Supplement: S7 Fig — Venn diagram of observed genera across treatments to highlight the direct colonization of A) peat and B) soil rhizospheres by vermicompost-origin taxa. The associated tables list genera corresponding to the groups delineated by the diagram. (TIF) [file pone.0230577.s007.tif]

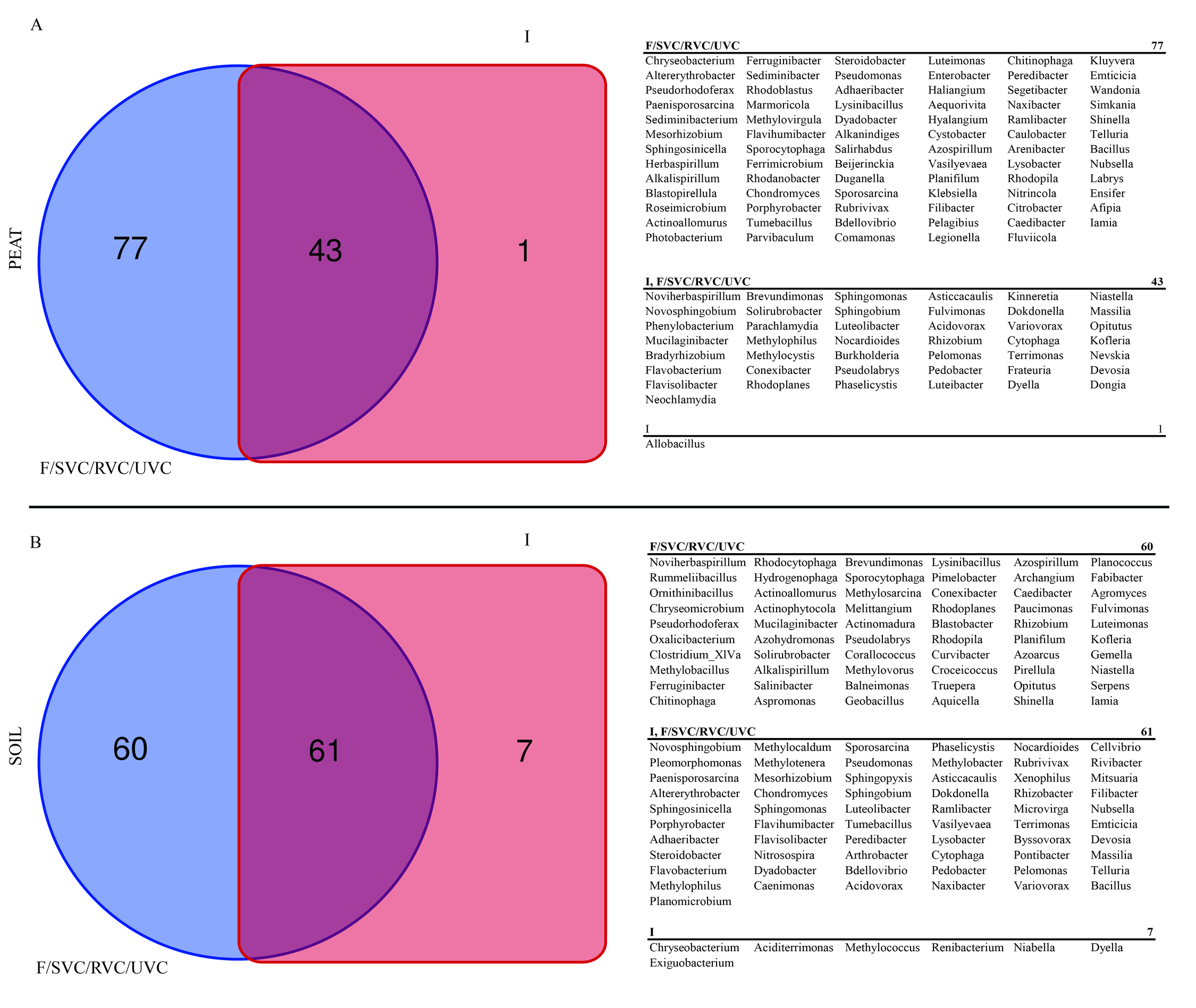

Supplement: S8 Fig — Venn diagram of observed genera across treatments to highlight potential general fertilization responses of A) peat and B) soil rhizospheres to VC treatments. The associated tables list genera corresponding to the groups delineated by the diagram. (TIF) [file pone.0230577.s008.tif]

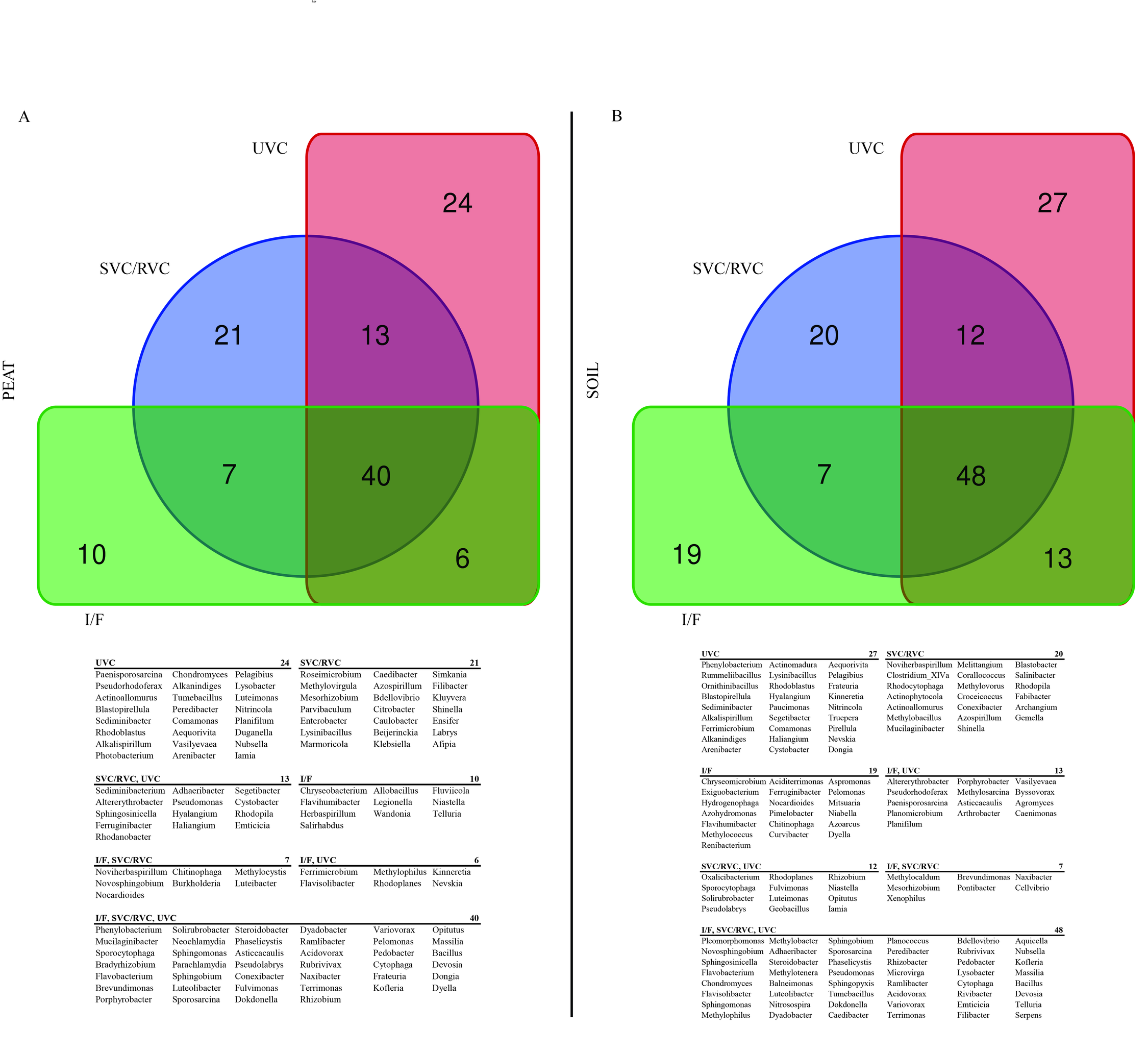

Supplement: S9 Fig — Venn diagram of observed genera across treatments to highlight potential autoclaving effects of A) peat and B) soil rhizospheres to VC treatments. The associated tables list genera corresponding to the groups delineated by the diagram. (TIF) [file pone.0230577.s009.tif]

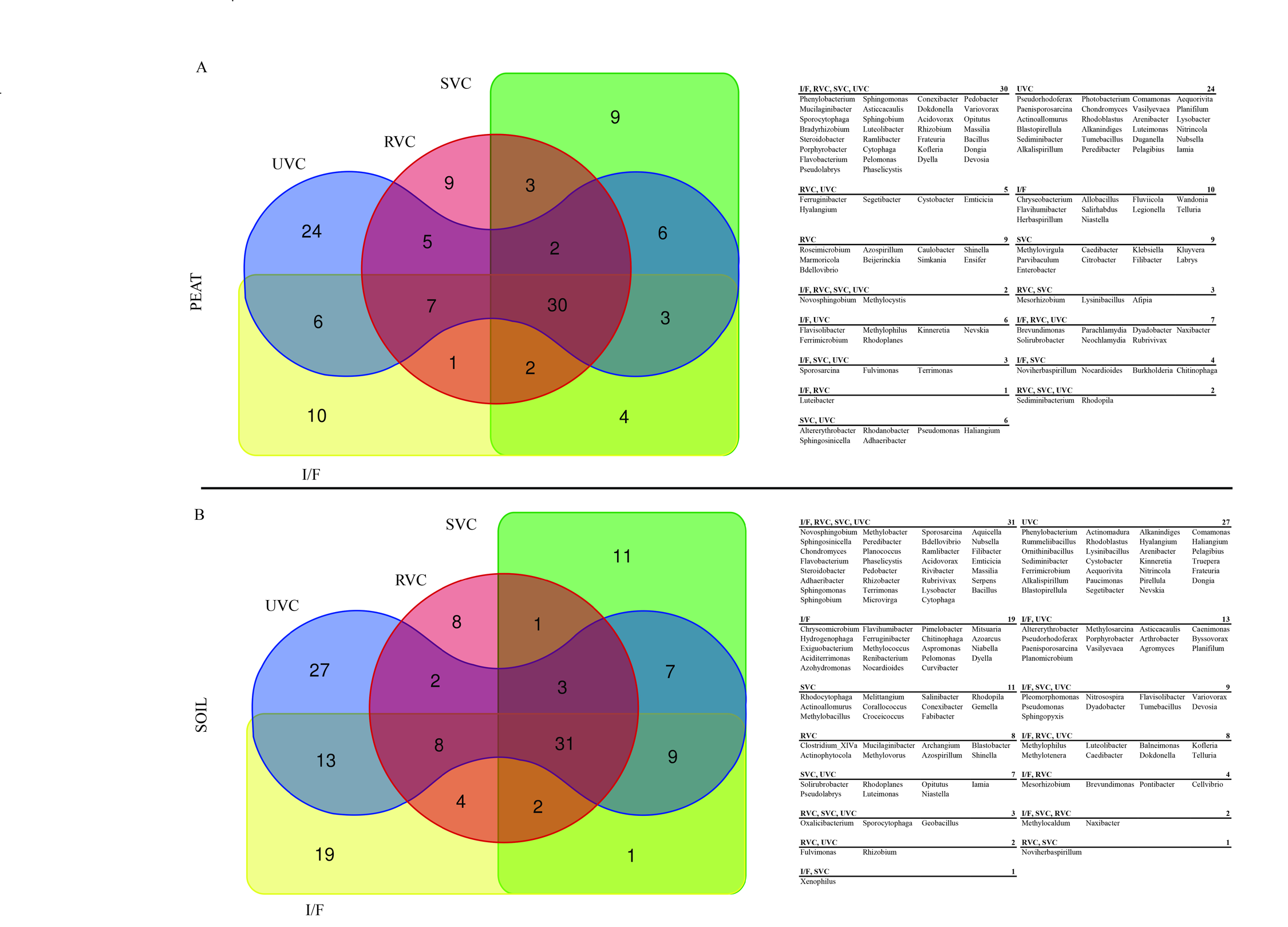

Supplement: S10 Fig — Venn diagram of observed genera across treatments to highlight potential direct and indirect biotic effects of A) peat and B) soil rhizospheres to VC treatments. The associated tables list genera corresponding to the groups delineated by the diagram. (TIF) [file pone.0230577.s010.tif]
